# Endodontic assessment, complexity, diagnosis and treatment planning

**DOI:** 10.1038/s41415-025-8452-6

**Published:** 2025-04-11

**Authors:** Obyda Essam, Shakil Umerji, Kate Blundell

**Affiliations:** Department of Restorative Dentistry, School of Dentistry, University of Liverpool, Liverpool, United Kingdom

## Abstract

A systematic approach to endodontic clinical assessment, case complexity evaluation and precise diagnosis is essential for achieving successful endodontic management and favourable treatment outcomes. Neglecting these foundational elements can jeopardise the entire treatment process. This article is part of a themed issue dedicated to endodontics and serves as a practical guide for clinicians, outlining the comprehensive process of endodontic assessment. It will encompass clinical examination, radiographic interpretation and diagnostic testing, ultimately providing clinicians with the necessary tools for accurate diagnosis and effective treatment planning.

## Key points

- Provides a practical guide and a step-by-step approach to endodontic assessment, improving diagnostic accuracy and treatment planning.
- Discusses the benefits and limitations of endodontic case complexity assessment tools, aiding in efficient case selection and referral decisions.
- Offers a holistic perspective and integrates various treatment and complexity factors influencing endodontic treatment success.
- Highlights the importance of informed consent and clear patient communication.

## Introduction

Systematic endodontic clinical assessment, case complexity assessment and accurate diagnosis are the cornerstones of successful endodontic management and favourable treatment outcomes. Failing to establish these foundations can compromise the entire process. This article is a practical guide for clinicians, detailing the process of endodontic assessment. It will cover clinical examination, radiographic interpretation and diagnostic testing, ultimately equipping clinicians with the tools for accurate diagnosis and informed treatment planning.

Endodontic pathology manifests as a spectrum of signs and symptoms, varying from incidental findings during routine examinations, often unnoticed by the patient, to cases where patients present with acute distress and significant discomfort.

## Capturing clinical history

It is crucial to establish a strong rapport from the outset. Engaging with a distressed patient requires empathy, alongside a clear focus on acquiring essential information regarding their presenting symptoms. Open-ended questions encourage the patient to express their concerns freely. The British Endodontic Society's *A guide to good endodontic practice* suggests that the use of the mnemonic ‘SOCRATES' may be particularly helpful in establishing a history:^1^

- Site - where is the pain located?
- Onset - when did the pain start?
- Character - what kind of pain is it? Sharp, dull, throbbing?
- Radiation - does the pain spread to other areas?
- Associations - what other symptoms occur alongside the pain?
- Time course - does the pain change over time? Does the pain disturb your sleep?
- Exacerbating/relieving factors - what makes the pain worse or better?
- Severity - how severe is the pain on a scale of 1-10?

Gathering an accurate history is crucial to establishing an accurate diagnosis and effective treatment planning; however, it is essential to recognise that relying solely on pre-operative symptoms for definitive treatments may be inappropriate. The diagnosis section later discusses more recent attempts to link the clinical diagnosis to the histological status.

## Clinical examination

Starting with an extra-oral examination, the clinician can assess for the presence of facial swelling or asymmetry, being mindful of the potential for systemic spread of dental infection (eg cellulitis); although, such cases are thankfully uncommon. Critical indicators of systemic involvement include pyrexia and difficulty swallowing or breathing, which may signify airway restriction that requires urgent hospital referral. Additionally, palpation of the temporomandibular joint (TMJ) and lymph nodes is essential. TMJ dysfunction should be ruled out in cases of pain, which may mimic endodontic issues.

The intra-oral examination aims to identify the potential causes of the patient's symptoms. It begins with assessing the patient's general dental hygiene status and examining the oral soft tissues. It is the responsibility of any clinician who examines the patient to be able to identify and report abnormalities. Dental professionals should be vigilant in recognising soft tissue irregularities and arrange for further investigation or referral when needed.

When assessing the area of concern, the clinician should gently palpate the mucosa surrounding the tooth to assess for any tenderness that may indicate inflammatory changes, such as severe or irreversible pulpitis, apical periodontitis or associated swelling. Key aspects to evaluate include the contour and texture of the tissues, such as observing for firmness or fluctuation, and noting any redness or yellowing of the tissue. Additionally, the clinician should check for fistulas or discharge from soft tissues.

**Fig. 1:**
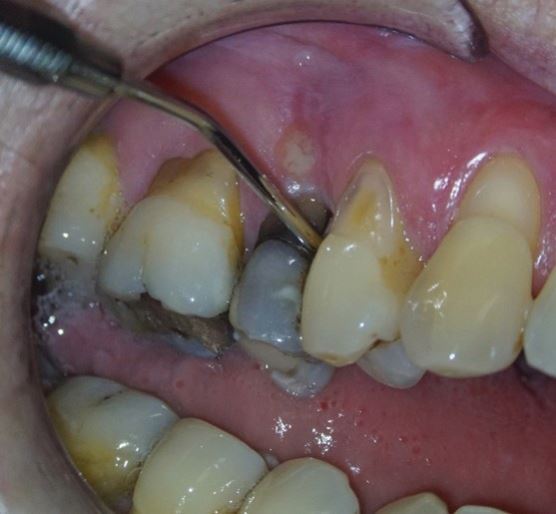
Deep isolated pocket related to vertical root fracture

A screening assessment for periodontitis is vital to assess the general periodontal condition. If deeper pockets (corresponding to Basic Periodontal Examination (BPE) code 3 or 4) are identified, a comprehensive periodontal evaluation should be advised, recording the pocket depths at six points around the tooth to be treated. Isolated and localised periodontal pockets around the concerned tooth may signify an endodontic complication, such as vertical root fractures (VRF) (Fig. 1), root perforations, or a periodontally draining fistula tract, indicating that the periodontal condition could be secondary to endodontic pathology. However, it's important to highlight that this is more of a generalisation and that an isolated pocket may not always be present or detectable. A periodontal pocket is generally formed due to the bony destruction during the progression of a VRF. However, it is important to remember that is not always the case, as no osseous defect or deep probing depth may be evident in the early stages.^2^ The presence of tooth mobility, furcation involvement or recession should also be noted if present.

Careful evaluation and documentation of the condition of the tooth is essential. This involves assessing how much coronal tooth structure remains, evaluating the state of any existing restorations and looking for any fractures or cracks. It's also crucial to note any discolouration and whether the tooth shows signs of fremitus or tenderness to percussion.

Endodontic treatment may be indicated when there is a significant loss of coronal tooth structure due to caries or trauma, so assessing whether the tooth can be restored before proceeding is essential. For restoratively predictable results, there should ideally be more than 2 mm of circumferential supra-gingival tooth structure and more than 30% of the original coronal tooth structure remaining. The Dental Practicality Index was developed to assess the feasibility of restoring a tooth and referring it for additional care or extraction.^3^ An occlusal examination should also be conducted early to determine the occlusal load for future tooth restoration. In cases involving a mixed dentition, the positions of both erupted and unerupted teeth, alongside the condition and prognosis of any questionable deciduous or permanent teeth, should be evaluated.

Sensibility tests are valuable assets for endodontic assessment. These include thermal tests, as well as electric pulp testers (EPT). Traditionally, ethyl chloride (with a temperature of around -5 °C) has been used for cold testing for years, but more recently, hydrochlorofluorocarbon refrigerant sprays, such as tetrafluoroethane, which operate at temperatures below -25 °C, are considered more accurate and should routinely be used instead.

Although cold tests are often more practical and reliable for clinical assessment,^3^ combining cold tests and EPT, and clinical signs and symptoms, is recommended to enhance diagnostic accuracy. This integrated approach facilitates more informed treatment decisions. In a systematic review,^3^ the probability of eliciting a true positive reaction representing a vital pulp was highest with a cold test, while it may generate lower accuracy with teeth exhibiting calcified canals, where the EPT may be more helpful. Reviews often address the sensitivity (ability to correctly identify vital pulp) and specificity (ability to correctly identify non-vital pulp) of EPT. Some studies suggest that while EPT has high specificity, its sensitivity is variable, leading to the recommendation of using it alongside other tests. Combining both tests with the clinical signs and symptoms can enhance diagnostic accuracy and inform treatment decisions. In some cases, using a heat test using warm water in a syringe with a dental dam in place or warm gutta-percha points may also be helpful, but this may not be as practical in clinical practice.

Selective anaesthesia can also assist in localising symptoms; for instance, an inferior dental block may help differentiate poorly localised pain in the upper or lower jaw, while local infiltration can effectively isolate a suspected tooth within a specific quadrant.

Other methods reported to help assess the pulp vitality include laser doppler flowmetry and pulse oximetry.^4^ Both represent helpful advancements in pulp vitality assessment; however, these are less widely accessible in clinical practice at this stage.

## Radiographic examination

Radiographic evaluation is essential for diagnosing suspected issues following a clinical examination. For endodontic assessments, periapical radiographs (PAs) are typically the preferred first-line imaging choice, usually obtained using a beam-aiming device. PA radiographs are valuable for identifying periapical lesions and signs indicative of bone resorption around the apex, as well as for detecting internal and external root resorption. Root resorption is explored in greater detail in a separate article in this series.

Periapical radiolucencies can signify a range of conditions originating from both odontogenic and non-odontogenic sources; however, a comprehensive discussion of these is beyond the scope of this article. Nevertheless, it is crucial to consider non-odontogenic causes when interpreting radiographs, as this awareness can influence diagnostic accuracy and subsequent treatment decisions. From an endodontic perspective, the most prevalent presentations for these lesions are periapical granulomas, abscesses and radicular cysts. A periapical granuloma is a chronic lesion that develops due to a periapical inflammation characterised by granulation tissue. A periapical abscess is defined by the accumulation of pus within the periapical tissue, often presenting with a swelling (acute abscess) or a draining fistula (chronic abscess). In contrast, a periapical cyst is a well-defined, epithelium-lined, fluid-filled cavity, commonly associated with larger, long-standing chronic periapical periodontitis, frequently exhibiting signs of bone cortication. The size and shape of the lesion provide important diagnostic clues. Cysts are usually well-defined and round with a regular border, while abscesses and granulomas may appear ill-defined and irregular.

Additionally, PA radiographs can reveal the presence and extent of caries, assess restorations, identify pulp stones and evaluate the pulp chamber size. Additionally, PAs help detect the widening of the periodontal ligament space, loss of the lamina dura, apical and lateral radiolucencies, and bone loss due to perforations or root fractures. Taking two or more PAs at different angles (parallax) is recommended to identify unusual anatomy or superimposed pathology. Changing angles can help visualise conditions that may be hidden in one plane.

It's important to note that endodontic lesions typically become detectable on PAs only after they reach the cortical and cancellous bone junction.^5^ While there is a correlation between the status of periradicular tissues and the presence of an apical radiolucency, its absence does not necessarily indicate no pathology.

If available, bitewing radiographs may also provide valuable insights into root morphology and pulp chamber position. They allow clinicians to visualise the alignment and morphology of the roots, especially in multirooted teeth, and offer information about the depth and location of the pulp chamber relative to the coronal structure, aiding in access during root canal treatment. Bitewings may also suggest variations in canal anatomy, prompting further investigation.

In some instances, where two-dimensional radiography yields inconclusive results, cone beam computed tomography (CBCT) is increasingly used in endodontics due to its ability to provide detailed three-dimensional imaging that enhances diagnostic accuracy, especially in cases involving pathological root resorption, trauma, unusual anatomy, or surgical treatment planning. The application of CBCT in endodontics is explored in greater detail in another article in this series.

## Diagnosis

A definitive or provisional diagnosis should be made after carefully considering the patient's symptoms, clinical examination findings and test results. It is helpful to state both pulpal and periodontal diagnoses of the tooth when documenting the outcome. The pulpal diagnosis should include the status of the dental pulp, indicating whether it is vital, non-vital, or inflamed, while the periodontal diagnosis relates to the state of the periapical or radicular periodontium.

Table 1 (from the British Endodontic Society's *Guide to Good Endodontic Practice*)^1^ is based on the current terminology for endodontic diagnosis as defined by the guidelines of the American Association of Endodontists (AAE). The term ‘apical' is sometimes described as ‘periapical' or ‘periradicular' in other literature. For clarity and consistency, we have decided to use the same terminology the British Endodontic Society used in Table 1.

**Table 1:** Summary of diagnostic terminology with associated symptoms, signs and radiographic appearance. Reproduced with permission from the British Endodontic Society, *A guide to good endodontic practice*, 2022^1^

**Diagnosis**	**Symptoms or signs**	**Radiographic feature**
Reversible pulpitis	Short, sharp sensitivity to cold Localised to the affected tooth Questionable restoration, coronal fracture	Normal appearance of apical tissues, often intact lamina dura
Irreversible pulpitis	Spontaneous pain, lingering, intermittent, poorly localised to a specific tooth Can radiate away from the source Pain may refer to adjacent teeth, face, jaw, temporal regions	Normal apical tissues but may show early signs of apical involvement if longstanding eg widening of the periodontal ligament (PDL) space, loss of lamina dura
Pulp necrosis	None, or vague history of intermittent symptoms which ultimately settle	Normal periapical tissues but may show early signs of apical involvement if longstanding eg widening of the PDL space, loss of lamina dura
Transient apical periodontitis (eg occlusal trauma, dental trauma)	Percussive tenderness of the affected tooth Similar to reversible pulpitis: thermal hypersensitivity of short duration	Intact lamina dura Widening of the PDL space around the root in response to excessive occlusal forces or interference
Symptomatic apical periodontitis	Tenderness to percussion Tenderness upon palpation of periapical soft/hard tissues of the affected tooth Sporadic pain, odd ‘niggle'	Widening of the PDL space Loss of lamina dura
Asymptomatic apical periodontitis	No symptoms Sometimes small, firm (bony) swelling over the apex of affected tooth	Rarefaction of apical bone architecture around roots (radiolucency)
Chronic apical abscess	Little or no symptoms Discharging suppurating sinus	Rarefaction of apical bone architecture around roots (radiolucency)
Acute apical abscess	Severe pain and swelling Intra-oral: often fluctuant Extra-oral: more diffuse progressing to cellulitis	Typically little or no noticeable radiographic changes (unless the condition is an acute exacerbation of existing apical periodontitis)
Condensing osteitis	May or may not be symptomatic May be associated with either symptomatic or asymptomatic apical periodontitis	Diffuse radiopaque lesion representing a localised bony reaction to a low-grade inflammatory stimulus

While a generalisation, poorly localised pain triggered by thermal sensitivity may indicate symptoms related to pulpitis. If the pain is severe and primarily associated with hot stimuli, lingers over time, or occurs spontaneously without any apparent trigger, it is more likely indicative of irreversible pulpitis. Late-stage signs of irreversible pulpitis may include tenderness when biting or palpating the adjacent soft tissue.

In contrast, well-localised, constant, throbbing pain, unaffected by thermal stimuli is generally more associated with periodontitis of the periapical or periradicular tissues. This is often accompanied by severe sensitivity to biting, the sensation of the tooth feeling elevated and may also be associated with swelling.

As alluded to earlier, while achieving an accurate diagnosis is vital for treatment planning, definitive treatment planning based on pre-operative symptoms alone may be inappropriate. Such diagnoses are usually made for clinical convenience, as it is impossible to determine the pulp's histological status clinically without tooth extraction and histological analysis. While categorising pulpitis as ‘reversible' or ‘irreversible' remains useful, a patient's symptoms and clinical appearance may not fit into either category.

A positive advancement in pulpal diagnosis was the introduction of a classification based on clinical symptoms.^6^ This classified pulpitis as:

- Mild reversible pulpitis - patients describe sensitivity to hot and cold, lasting up to 15-20 seconds, and settling spontaneously
- Severe reversible pulpitis - increased pain for more than several minutes and needing oral analgesics
- Irreversible pulpitis - persistent dull throbbing pain, sharp, spontaneous pain and tenderness to percussion. Pain is possibly exacerbated by lying flat.

More recently proposed classifications attempt to recognise that the various stages of inflammation are much more of a continuum. Table 2 is adapted from an excellent article published in the *British Dental Journal*^7^ based on the proposed diagnostic system suggested by Wolters *et al.*^8^

**Table 2:** Proposed diagnostic criteria for pulpitis. Reproduced with permission from Edwards *et al.*, ‘Preserving pulp vitality: part two - vital pulp therapies', *British Dental Journal*, vol 230, pp 148-155, 2021, Springer Nature^7^^,^^8^

**Diagnosis**	**Likely symptoms**	**Likely histology**	**Proposed treatment**
Initial pulpitis	Heightened but not lingering response to cold Not tender to percussion (TTP) No spontaneous pain	‘Healthy pulp' or local inflammation confined to the coronal pulp	Indirect pulp therapy/coronal pulpotomy
Mild Pulpitis	Heightened and lingering response to cold, warm and sweet stimuli, which can last up to 20 seconds Possibly TTP	Local inflammation confined to the coronal pulp	Indirect pulp therapy/coronal pulpotomy
Moderate pulpitis	Strong, heightened and prolonged reaction to cold, which can last for minutes Possibly TTP Possible spontaneous dull pain, which can controlled by analgesics	Extensive local inflammation confined to the coronal pulp	Partial/complete pulpotomy
Severe pulpitis	Severe spontaneous pain Clear pain reaction to cold/hot stimulus Often sharp to dull throbbing pain Sleep-affected TTP	Extensive local inflammation of the coronal pulp, which may extend beyond the coronal pulp into the root	Direct clinical visualisation of the pulp is advised if: No prolonged bleeding of radicular pulp stumps: coronal pulpotomy Continued bleeding: superficial pulpotomy with endodontics up to healthy pulp or complete pulpectomy (root canal treatment)

Vital pulp therapy, which includes partial and complete pulpotomy, is discussed in greater detail in another article in this journal's endodontic series.

## Complexity assessment of endodontic cases

Endodontics is a highly technical treatment modality, and cases may present with a wide variation in complexity. Pre-emptively identifying cases with increased difficulty can mitigate the risk of complications and adverse outcomes.^9^^,^^10^^,^^11^

Practitioners should determine which cases are within their scope of practice and treat or refer accordingly. However, this judgement can be rather subjective, making appropriate case selection a challenge.

**Fig. 2:**
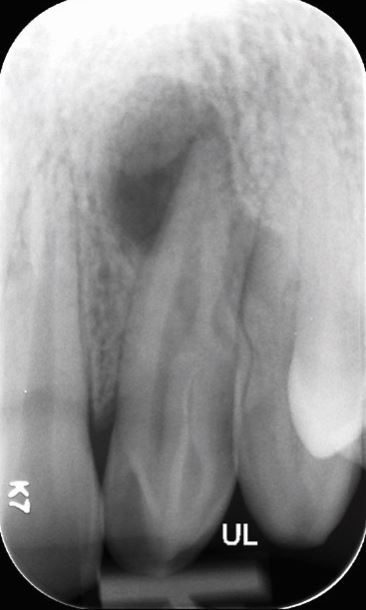
Tooth 22 with dens invaginatus (Oehlers Class IIIa)

**Fig. 3:**
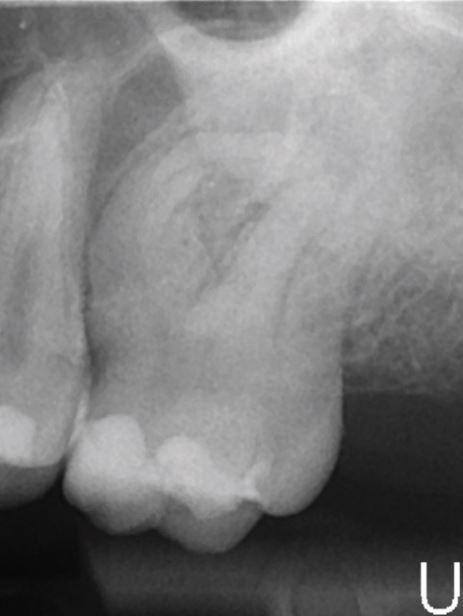
Extremely severe root curvature of tooth 16 mesiobuccal root

**Fig. 4:**
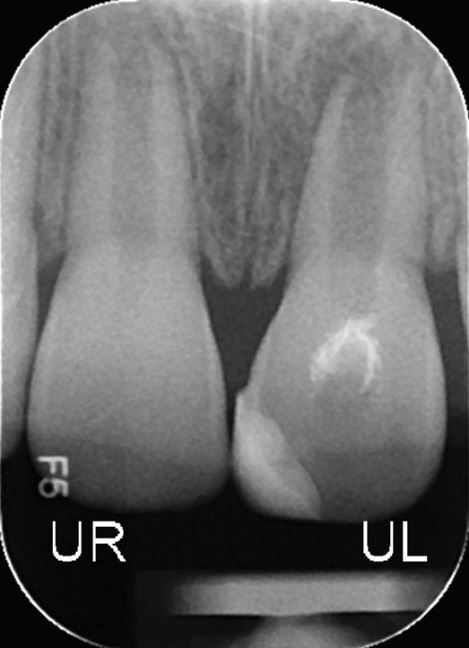
A traumatised tooth 21 with immature root development, resulting in an open apex

**Fig. 5:**
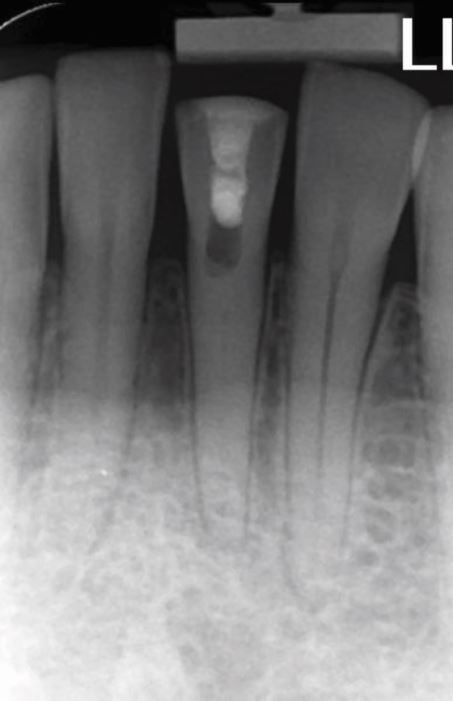
Tooth 41 with reduced canal visibility and previously initiated endodontic treatment

A range of factors have been recognised to influence the relative complexity of an endodontic case (Table 3, Fig. 2, 3, 4 and 5).^12^ These may be categorised into general patient-related factors (eg medical history) and tooth-specific factors (eg root curvature). Tooth-specific factors may be further divided based on clinical history, tooth positioning, periodontal involvement, coronal/restorative factors and finally, endodontic factors.

**Table 3:** General, patient-specific and tooth-specific complexity factors that should be considered when assessing case complexity^18^

**Factor type**	**Factor**
General/patient-related factors	Medical history Physical and psychological limitations Mouth opening Radiographic difficulties Complex diagnosis
Tooth-specific factors	History of trauma The position of the tooth Inclination and rotation of tooth Pre-treatment before commencement Crown morphology and presence of extra-coronal restoration (crown or overlay) (Fig. 2) Access to root canal system Root curvature (Fig. 3) Root canal morphology Apical morphology (Fig. 4) Canal radiographic visibility (Fig. 5) Previous endodontic treatment Iatrogenic incidents Root resorption Perio-endo (periodontic-endodontic) lesion involvement

Case difficulty assessment tools are available to systematically evaluate and collate these factors to grade complexity. Various tools are available, including the Restorative Dentistry Index of Treatment Need (RDITN) and case difficulty assessment forms by the Canadian, Dutch and American Endodontic Associations.^13^^,^^14^^,^^15^^,^^16^ Guidance is also available in the *Clinical standard for restorative dentistry* document for NHS care delivered in the United Kingdom.^17^

**Fig. 6:**
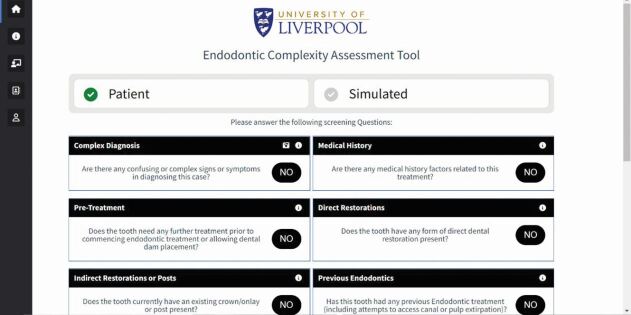
The E-CAT (www.e-cat.uk)^18^

**Fig. 7:**
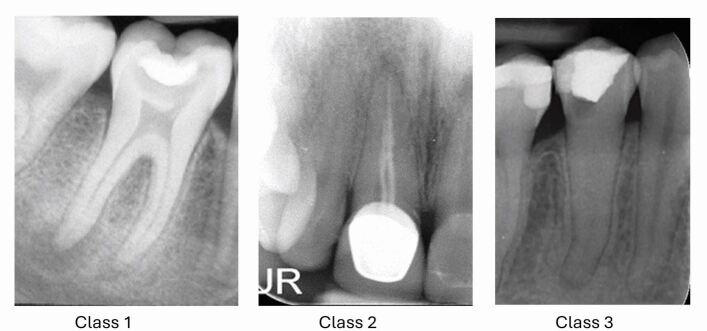
Examples of Class 1, 2 and 3 complexity cases

Contemporary digital assessment tools are also now available in the form of mobile and web-based applications. These include the Endodontic Complexity Assessment Tool (E-CAT) (Figure 6), which was partly funded by the European Society of Endodontology,^18^ the British Endodontic Society's ‘EndoApp',^19^ and the American Association of Endodontology's ‘AAE EndoCase' app.^20^ The tools all classify cases into three levels of difficulty (Figure 7) based on the risk for procedural errors and untoward outcomes, and are deemed suitable for management by three tiers of clinical experience, respectively (Table 4).

**Table 4:** Complexity grading used within case difficulty assessment tools, their associated risks and recommendations^18^

**Complexity grade**	**Risk**	**Triaging**
Class 1 (mild complexity)	Low risk of adverse events	Recent graduates (and undergraduates) or dentists with limited endodontic experience
Class 2 (moderate complexity)	Moderate risk of complications or compromised outcome	Practitioners with enhanced clinical experience or postgraduate training in endodontics
Class 3 (high complexity)	Higher risk of complications or compromised outcome	Specialist endodontic practitioners

A single highly complex factor or an accumulation of multiple lower complexity factors would increase the difficulty grade. The complexity level would correlate with the clinical expertise required and chair time expected, as well as the potential risks and challenges involved in treating the case.

The accuracy of case assessment tools depends on an individual's interpretation of a case and the user's correct utilisation of the tool. Therefore, there will always be potential for human error.^9^ However, when used correctly, they provide an objective and useful tool for endodontic treatment planning.

It is important to assess the complexity of a case and evaluate the challenges expected as part of a comprehensive consent process, as well as to inform appropriate management by a suitably experienced practitioner. Inappropriate case selection may lead to complications detrimental to patient outcomes and potential litigation. Pre-empting the challenges involved in treating a case and identifying when to refer can mitigate the risks involved in endodontics.

**Fig. 8:**
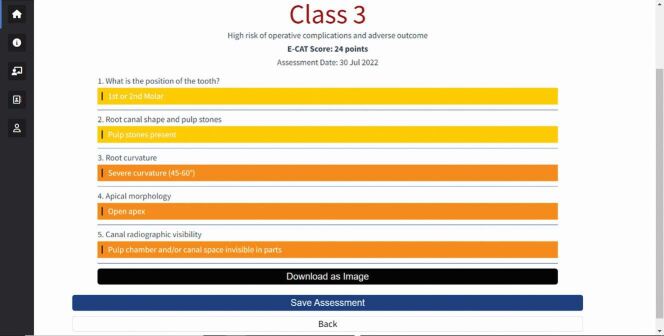
An example of a case assessment summary generated by E-CAT^18^

Moreover, an objective assessment summary (Figure 8) can be used not only for treatment planning, but also to assist primary care practitioners in effectively communicating and justifying the need for a referral to patients. Assessments could also be retained in clinical records to demonstrate that clinicians are working within their competency.

In addition, using case assessment tools for complexity-based triaging could improve access to primary care and referral services. Other benefits may be realised in universities, including a standardised and improved learning experience for undergraduate and postgraduate students, as well as utilisation in endodontic research.^9^

## Treatment planning in endodontics

The objectives of treatment planning in endodontics are to ensure the successful treatment of dental pulp and/or periapical tissues, where either become diseased, aiming to maintain the natural tooth and restore its function.^21^

Once the pulpal and periodontal diagnoses are established, this can be followed by a comprehensive discussion about the options for treatment, which may include: no active treatment; vital pulp therapy; endodontic treatment; re-treatment; endodontic surgery; or tooth extraction. If a decision is made not to initiate treatment, a period of watchful waiting with clinical and radiographic assessment may help the clinician to decide whether, for example, an asymptomatic periapical (peri-radicular) radiolucency is resolving, quiescent, or progressing.^22^

Medical history must be carefully considered as part of a holistic treatment plan, for example, a patient who is due to undergo chemotherapy may require a much more pragmatic approach than a patient who is fit and well. It may be important to consider whether endodontics is appropriate at all before treatment, or whether removal of the offending tooth may be in the patient's best interests if there is a risk of sepsis, for example. The timing of treatment between courses of drug treatment (chemotherapy) may require liaison with other medical colleagues and a referral to secondary care may be appropriate in the case of patients with complex medical needs. Conditions such as diabetes, or corticosteriod therapy, may impact the patient's immune response and healing capacity, which ultimately may impact on tooth survival post-endodontics.^23^ Oral and intravenous bisphosphonates are often seen in patients with a history of osteoporosis and/or metastatic cancer. These drugs may adversely affect bone healing quite significantly, depending on the type, the route of administration and the length of time they have been taken. For patients who have been prescribed intravenous bisphosphonates for the prevention of metastases following a cancer diagnosis and/or treatment, the dose would be much greater than that given for osteoporosis. In some cases, retaining teeth or roots at the gingival level may be advantageous, even if structurally compromised, to avoid the need for extraction and the risk of medication-related osteonecrosis of the jaw.^24^^,^^25^

As discussed earlier in this paper, several complexity assessment tools may be used to assess and judge whether the treatment may be outside the scope of the treating clinician's skill set. In such cases, a referral to a specialist endodontist may be indicated. In particularly complex cases, deciding to refer early may be in the patient's best interests.

There are some unknowns that we face as clinicians and sometimes it is not possible to say whether a tooth will be restorable without some degree of clinical investigation. In some cases, this may be beneficial to the patient - even if the outcome is that the treatment is not feasible, it may help eliminate the feelings of ‘what if'; however, this must be balanced against the costs of undertaking this investigatory work and patients must see it as an investment to gain knowledge or insight, rather than an expensive waste of both of your time. The treatment plan should also include provision for restoring the tooth to its proper function and appearance with appropriate restorative materials, considering the patient's occlusion and overall dental health status.

When planning the treatment, we must also consider patient preferences, lifestyle and financial situation. The care of patients in acute pain can pose significant challenges, especially when these patients are often ‘squeezed into' the appointment book, and in our world of digital dentistry, where information can be gathered very quickly, it can feel at times like an ‘instant diagnosis and solution' is required; however, ensuring that you make the time to consider all of the information to help you to formulate a diagnosis and provide the correct treatment can be difficult when there is a need to attain good anaesthesia and perhaps open a pulp chamber under dental dam. This type of treatment requires time and sometimes patience to be undertaken safely and effectively.

Managing patient expectations, especially where the prognosis is guarded, is vital to building trust and rapport, and honesty and transparency should be at the heart of any discussion with patients.

Our role is to help the patient decide the best option for them, weighing up the options alongside their respective risks and benefits and the likelihood of success of each option where possible. The decision ultimately lies with the patient. It must be borne in mind that success cannot be guaranteed and what we attempt to do in endodontics is to create an environment that favours a healing response. However, the propensity for healing is variable in the treatment process. We cannot ascertain how the body will respond to treatment as numerous factors can affect the outcome, which cannot be controlled in the dental clinic. Planning for follow-up appointments allows the treating clinician to monitor healing and address any issues promptly.

We have a duty to ensure the patient fully understands the proposed treatments, including potential outcomes and complications. Patients should be informed about the procedure, postoperative care and any potential complications that may arise in the short- and long-term where possible. Thus, informed consent must be obtained before proceeding with treatment.

## Conclusion

This paper outlines the various aspects to consider in a comprehensive endodontic assessment and the numerous factors that can complicate endodontic treatment, highlighting the importance of thorough case assessment and selection. Endodontic patients may present with a range of symptoms, from asymptomatic findings to those in acute distress requiring urgent care. A detailed patient history and systematic examination are essential for accurate diagnosis, emphasising the necessity of a structured approach.

The clinical case assessment and complexity tools to assist clinicians in deciding whether to manage a case or refer to a specialist are discussed. This also facilitates discussions about treatment options, prognosis, potential complications and a provisional plan for definitive restoration. Open and transparent communication regarding treatment challenges and possible complications builds trust and empowers patients to make informed decisions about their care.
